# A Reversible Cause of Complete Heart Block Causing Chest Pain and Syncope

**DOI:** 10.7759/cureus.1953

**Published:** 2017-12-16

**Authors:** Syed Rafay Ali Sabzwari, Zoltan Varga, Khurram Butt, Nimra Khan

**Affiliations:** 1 Cardiology Fellowship, Lehigh valley health network; 2 Internal Medicine Residency, Florida Hospital-Orlando; 3 Medicine, Florida Hospital-Orlando

**Keywords:** complete heart block, vasospastic angina, syncope

## Abstract

Vasospastic angina (VSA), also known as variant or Prinzmetal’s angina, is a relatively uncommon cause of retrosternal chest pain with transient ST segment elevation, mainly due to vasospasm in the coronary arteries. This is a case of 37-year-old female who presented with chest pain and syncope. Her initial workup, including echocardiogram, was negative. Subsequently, she was sent home with an event monitor. During the next two weeks, she continued to have recurrent episodes of similar chest pains and presented to her cardiology appointment with a heart rate of 45 bpm and blood pressure of 100/60 mmHg and was taken to hospital emergency department. Event monitor review showed intermittent complete heart block. In the hospital, the electrocardiogram (EKG) showed complete heart block and inferior lead ST elevations concomitantly with the chest pains. Although suspicious for vasospastic angina, coronary artery disease had to be ruled out for which patient underwent coronary angiography without evidence of significant obstructive disease. Immediately thereafter, the patient underwent permanent pacemaker placement without recurrence of syncopal episodes. This case signifies complete heart block as one of the rare complications of vasospastic angina which otherwise can also lead to symptoms such as dizziness, shortness of breath, syncope, cardiac arrest, and sudden cardiac death.

## Introduction

Vasospastic angina (VSA), also known as variant or Prinzmetal’s angina, is a relatively uncommon cause of retrosternal chest pain with transient ST segment elevation, according to initial descriptions by Myron Prinzmetal in 1954 [1]. The cause of myocardial ischemia, in this case, seems to be vasospasm rather than obstruction by an atherosclerotic plaque or thrombus. Although the prognosis is better than in the case of thrombus-mediated acute ischemia, VSA can lead to polymorphic ventricular tachycardia, ventricular fibrillation with sudden cardiac death, or to life-threatening bradyarrhythmias, such as transient high-degree atrioventricular (AV) block or asystole with syncope [2-4]. We present a rare case of VSA, where complete heart block and subsequent syncope were the presenting complaints.

## Case presentation

A 37-year-old Caucasian female with past medical history of hypertension presented with syncope and chest pain. The pain was non-exertional, substernal, grabbing across the central chest, and associated with lightheadedness, dizziness, and shortness of breath occurring intermittently for the past few days. She had one episode of syncope lasting 15-20 seconds, sudden in onset, without pre or post-syncopal symptoms. Her father died from myocardial infarction at the age of 42, she smoked one pack per day for past 20 years, with occasional alcohol and no illicit drug use. Her only home medication was lisinopril 5 mg. Neurological and cardiovascular examinations were unremarkable. Workup for ischemia and syncope including electrocardiogram (EKG), serial troponins, urine drug screen, and nuclear stress test were negative. Echocardiogram revealed a normal ejection fraction and no structural heart disease. Due to unclear etiology of the first episode of syncope, she was sent home with event monitor for outpatient follow-up with cardiology.

**Figure 1:**
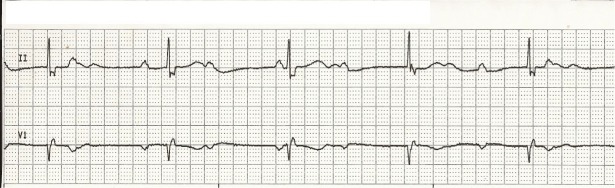
Telemetry strip showing complete heart block and junctional escape rhythm

**Figure 2:**
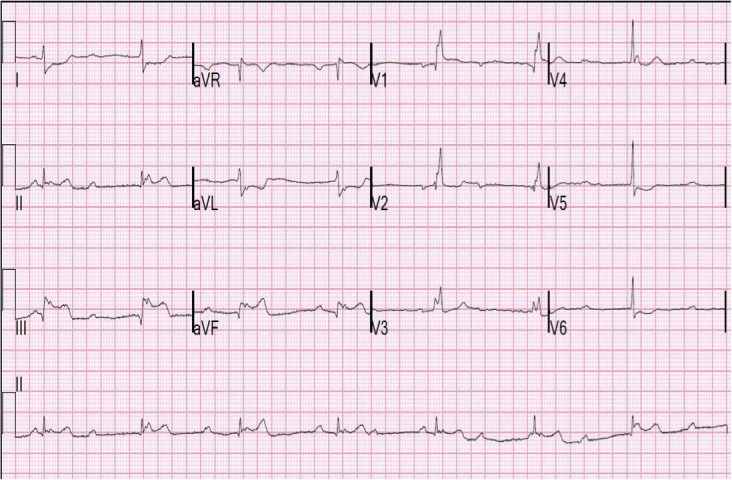
Electrocardiogram (EKG) showing complete heart block, junctional escape and inferior lead ST elevations

During the next two weeks, she continued to have recurrent episodes of similar chest pains and presented to her cardiology appointment with a heart rate of 45 bpm and blood pressure of 100/60 mmHg, feeling extremely dizzy and lightheaded, and was taken to the emergency department immediately. Troponins again were negative and the EKG did not show any new changes. Review of the event monitor, however, showed six episodes of 30-40 seconds of intermittent complete heart block. Overnight, the patient continued to have episodes of substernal chest pain, shortness of breath, and dizziness with telemetry showing third-degree heart block with junctional escape rhythm concomitantly with the chest pains (Figure 1). A twelve-lead EKG was performed which showed inferior lead ST elevations and third-degree heart block with junctional escape rhythm (Figure 2). Repeat troponin at this time was 0.04 ng/ml. Although this pointed more towards vasospastic angina, atherosclerotic coronary artery disease needed to be ruled out; therefore the patient was started on heparin and nitroglycerin infusion per acute coronary syndrome protocol. Subsequently, the patient underwent left heart catheterization that showed evidence of right coronary artery vasospasm and diffuse small vessel coronary artery disease but no isolated lesion to require intervention thereby confirming the diagnosis of vasospastic angina. Therefore, the same day patient underwent a dual chamber permanent pacemaker placement for symptomatic complete heart block and was started on isosorbide mononitrate. Statin could not be started due to her previously reported intolerance in the form of severe muscle aches. Over the next 24 hours, she had no further episodes of chest pain or lightheadedness but continued to have intermittent pacing on telemetry. On outpatient follow-up, she had no further complaints, and pacemaker interrogation showed 5% pacing in three months.

## Discussion

Prinzmetal angina or vasospastic angina is a disorder characterized by episodic chest pain that is induced by transient coronary artery vasospasm with or without coronary atherosclerosis. Prinzmetal angina leads to transient changes, the most common being ST elevations [5]. The populations affected are usually young females, often with a history of smoking. Other risk factors include the use of stimulant drugs, food-borne botulism, magnesium deficiency, and balloon dilatation during percutaneous coronary interventions. In 25% of cases, an episode of vasospastic angina can lead to arrhythmias, including atrioventricular block or supraventricular arrhythmias, ventricular extrasystoles, ventricular tachycardia, and ventricular fibrillation [6-8].

This case has the unique presentation of a young female who had an attack of vasospastic angina accompanied by intermittent complete heart block leading to chest pain and syncope. Complete heart block is one of the rare complications of vasospastic angina and can lead to symptoms like dizziness, shortness of breath, syncope, cardiac arrest, and sudden cardiac death. These patients can be put on Holter monitoring to diagnose the etiology of such symptoms. Diagnosis of a high degree heart block warrants the use of a pacemaker to prevent further episodes of heart block. Additionally, the treatment includes the use of calcium channel blockers, long-acting nitrates, and smoking cessation that can be challenging, especially in such cases. Beta-blockers should be avoided in these patients as they may precipitate an attack and can lead to syncope and transient AV block [9-10]. The overall prognosis is excellent, with almost 94% surival at five years with medical therapy alone. The absence of significant arrhythmias and significant coronary disease confer additionally favorable prognoses.

## Conclusions

It is believed that focal or diffuse smooth muscle hyperreactivity causing vasospasm induces ischemia and myocardial dysfunction that can manifest as conduction abnormalities. As distinguished from ST elevation, myocardial infarction can be challenging but the transient nature, rapid reversal of ST segment changes, responsiveness to calcium channel blockers, and nitroglycerin can prove to be helpful.
